# Ethnic Enclaves and Metabolic Syndrome in Chinese Immigrants in Philadelphia

**DOI:** 10.1007/s10903-025-01815-6

**Published:** 2025-11-20

**Authors:** Edgardo A. Hernandez, Daisy Rojas, Carolyn Y. Fang, Brian L. Egleston, Amy H. Auchincloss, Emily Walton, Marilyn Tseng

**Affiliations:** 1 Department of Kinesiology and Public Health, California Polytechnic State University, San Luis Obispo, CA, USA; 2 Cancer Prevention and Control Program, Fox Chase Cancer Center, Philadelphia, PA, USA; 3 Department of Biostatistics and Bioinformatics, Fox Chase Cancer Center, Philadelphia, PA, USA; 4 Department of Epidemiology and Biostatistics, School of Public Health, Drexel University, Philadelphia, PA, USA; 5 Department of Sociology, Dartmouth College, Hanover, NH, USA

**Keywords:** Asian americans, Chinese immigrants, Cardiometabolic risk, Ethnic density, Ethnic enclaves, Longitudinal study, Metabolic syndrome

## Abstract

Ethnic enclaves, neighborhoods with high ethnic concentrations, may have a protective effect on their residents’ health outcomes, but studies on their associations with cardiometabolic risk in Asian communities are inconsistent. We examined whether ethnic enclave residence was associated with metabolic syndrome (MetS) in a longitudinal sample of 516 Chinese immigrant adults in Philadelphia. Participants were recruited from three types of neighborhoods: established enclaves, emerging enclaves and non-enclave neighborhoods. At baseline (9/18 – 1/20) and follow-up (8/21 – 4/22), research staff conducted interviews and anthropometric and blood pressure measurements and collected fasting blood samples for glucose, triglycerides, and high-density lipoprotein levels. We used logistic regressions estimated by generalized estimating equations to estimate odds ratios (OR) for associations of enclave residence with MetS and its components, and differences in change over time in models stratified on neighborhood type. Overall, no consistent associations between enclave residence and MetS or MetS components emerged. Over an average follow-up of 2.6 years, the occurrence of MetS increased significantly in the overall sample. In stratified analyses, the increase was significant only among non-enclave residents, but interaction p-values indicated no significant differences across neighborhood type. Our findings suggest that ethnic enclaves are not ‘monolithically beneficial’. A more nuanced understanding of the resources that different kinds of enclaves offer and of how Chinese immigrants interact with these enclave resources is needed to inform and support effective investment in immigrant communities.

## Background

Asian Americans are the fastest growing immigrant group in the nation [1]. Within this group, Chinese immigrants make up the largest proportion [2], many of them living in ethnic enclaves, neighborhoods with high concentrations of other Chinese immigrants. Cardiometabolic risk factors among Asian Americans appear to increase with acculturation and length of US residence, possibly due to lifestyle changes such as dietary practices [3–5]. Stressors associated with migration and marginalization may also produce physiologic changes that increase cardiometabolic risk (CMR) [6].

## Conceptual Framework

Ethnic enclaves may have a protective effect on the health of Asian Americans [7, 8]. A possible mechanism is by helping preserve cultural behaviors, such as eating habits or lifestyles that are healthier than those practiced in the general population. Ethnic enclaves also provide physical and social spaces, such as community centers and churches, that buffer against social stressors [9, 10]. Both mechanisms may benefit not only enclave residents but also non-residents who visit enclaves from other neighborhoods.

Findings from the few studies on ethnic density and CMR in Asian communities are inconsistent [11–18]. Most included Asians as one heterogeneous group or focused only on body mass index (BMI) as the outcome of interest. Additionally, past studies neglected to examine the degree to which immigrants engage with enclave resources regardless of whether they live in an enclave or not [19]. Moreover, most prior studies define enclaves based on ethnic density without measuring how this is reflected in the co-ethnic built environment, such as Chinese businesses, service providers, and cultural centers. Enclaves may also have different effects depending on their recency of establishment and socio-historical context [20]. Research has shown, for example, differences in levels and function of social capital in *established enclaves*, with both high ethnic concentration and visible symbols of Chinese culture, versus *emerging enclaves*, more recent immigrant destinations with relatively high ethnic concentration but lacking most physical structures common to a Chinatown [21]. Further, earlier studies have documented differences in built environment resources between established and emerging enclaves in Philadelphia [9].

We investigated the association of ethnic enclave residence with CMR, measured as the presence of metabolic syndrome (MetS) and its components (elevated waist circumference, elevated triglycerides, reduced high-density lipoproteins (HDL-C), elevated blood pressure (BP), elevated fasting glucose), in a longitudinal study of Chinese immigrants in Philadelphia. We hypothesized that:

1. Enclave residence would be associated with lower odds of MetS. More specifically,
  - Enclave residents would have lower odds of MetS compared to non-enclave residents; here, we further distinguished between *established enclave* and *emerging enclave* residents;
  - MetS would increase less over time in enclave vs. non-enclave residents;
2. Greater engagement with enclaves, operationalized as the extent to which activities such as employment, shopping, and health care took place in enclaves, would be associated with lower odds of MetS.

## Methods

### Participants

From September 2018 to December 2019, we recruited 520 adult Chinese immigrants into a longitudinal study on neighborhoods and CMR through community organizations, events, businesses, chain referrals, and contacts within the Chinese community. Research staff screened interested participants for eligibility, obtained written consent to participate, oriented participants to study procedures, and scheduled appointments for interviews and data collection visits. Eligibility criteria included: (1) Chinese heritage; (2) migration from Asia at age ≥ 18 y; (3) age 35–65 y; and (4) residence in the Philadelphia region. Exclusion criteria included: (1) known, physician-diagnosed and/or treated clinical disease (diabetes, myocardial infarction, stroke, heart failure, cardiovascular procedures, cancer (except non-melanoma skin cancer)); (2) pregnancy or lactation; (3) current or planned (within 2 years) nursing home residence; and (4) impaired cognitive ability or inability to provide informed consent. The study was reviewed and approved by the Fox Chase Cancer Center Institutional Review Board, and all contact and informed consent documents were provided in English and Chinese.

**Fig. 1:**
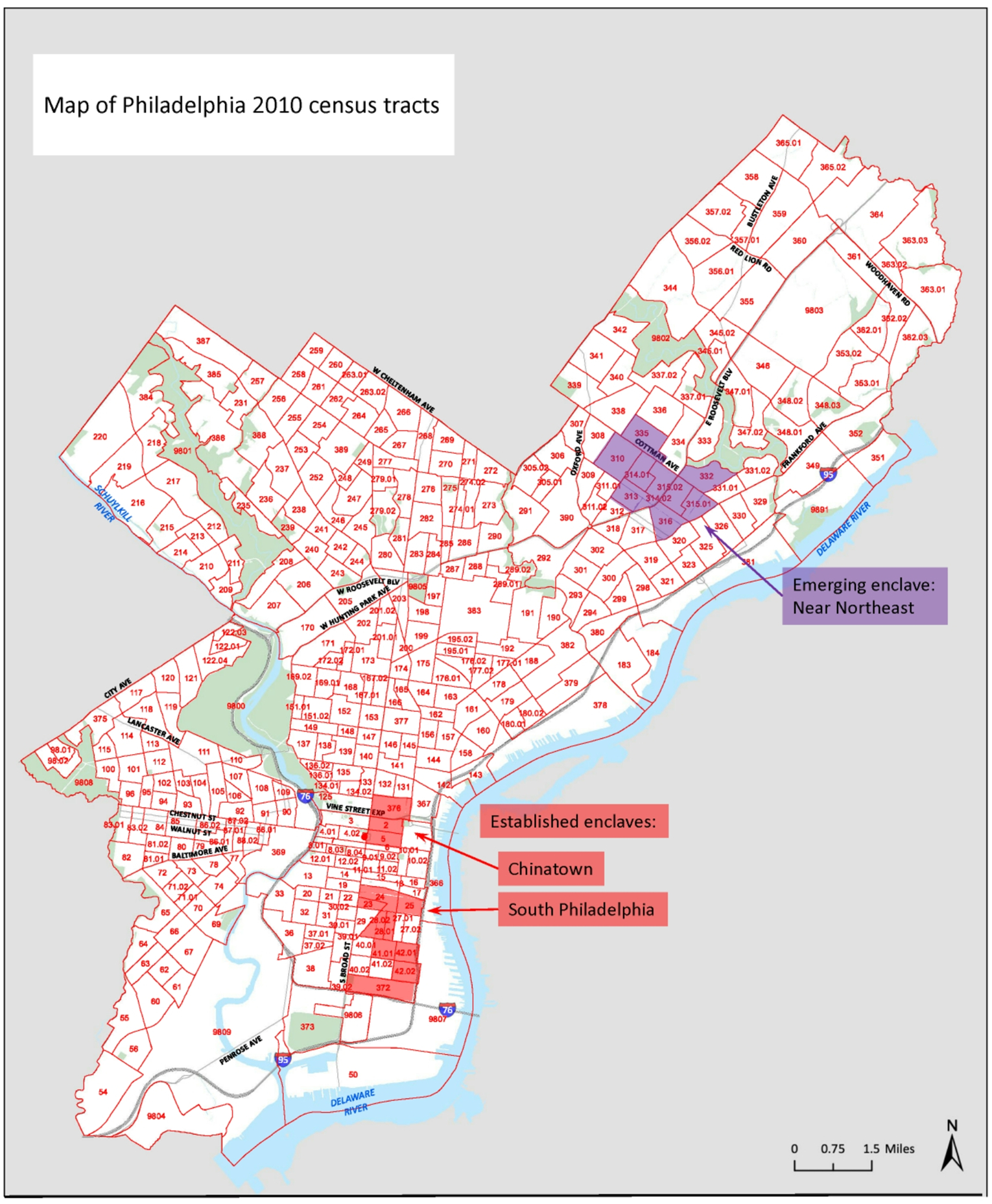
Census tracts included in established and emerging enclaves in Philadelphia [21]

We used a non-probability quota sampling approach to draw approximately equal proportions of the sample from each of 3 neighborhood types: established, emerging, and non-enclave. Established and emerging enclaves were identified through a systematic process using data from the American Community Survey (ACS) 5-Year Estimates (2014–2018). First, we calculated Location Quotients (LQ) as the ratio of the proportion of Chinese residents in a given census tract to the proportion of Chinese residents in the total population of the Philadelphia metropolitan area [21]. A z-score cutpoint of > 2.58 SD above the mean for the Philadelphia metropolitan area identified four areas. We then used our research team’s local knowledge (all interviewers reside in Philadelphia or its surrounding region and are familiar with local Chinese resources) and academic and lay sources [22–26] to select three of the areas recognized locally as Chinese enclaves, excluding one university neighborhood that included high proportions of Asian students. We distinguished ‘established’ and ‘emerging’ enclaves by examining change in LQ between 2000 and 2010. The established enclaves, in Center City (Chinatown) and South Philadelphia, were areas where at least one census tract maintained an LQ z-score > 2.58 in both 2000 and 2010. Census tracts in the emerging enclave in Philadelphia’s Near Northeast all had LQ z-scores < 1.96 in 2000 but > 2.58 in 2010 (Fig. 1). All other participants, residing in neighborhoods lacking the ethnic density and resources of recognized enclaves, were categorized as living in non-enclave neighborhoods.

### Data Collection

From September 2018 to January 2020, trained interviewers conducted anthropometry, blood pressure measurements, sample collections, and detailed baseline interviews in the appropriate dialect (Mandarin or Cantonese). Interviews elicited information on sociodemographic characteristics and other relevant variables. Waist circumference was measured in duplicate, and the mean used in analyses. Seated BP was measured after a five-minute rest in duplicate at one-minute intervals, and the mean used in analysis.

Study staff collected whole blood in one 10 mL EDTA (lavender-top) tube and serum in two serum-separator (gold-top) tubes after an overnight fast. Samples were labeled with a study ID and stored in a cooler with ice packs (2–8°C) until delivery to the FCCC Biosample Repository Facility for processing and storage at −80°C until analysis. Samples were transported locally to Quest and tested using standard clinical laboratory methods for glucose (spectrophotometry). The FCCC Clinical Laboratory performed assays for HDL-C and triglycerides with an Ortho Clinical Diagnostics Vitros Fusion 5,1 analyzer using conventional enzymatic colorimetric methods according to standardized clinical protocol.

Follow-up assessments were performed about two years after initial data collection using the same procedures. From August 2021 to April 2022, 416 participants were successfully re-contacted for follow-up, for a mean (SD) length of follow-up of 2.6 (0.4) years (range 1.7–3.4 years).

### Measures

We categorized respondents by neighborhood type (established, emerging, or non-enclave) based on residential address at recruitment. At baseline and follow-up, participants also indicated the address or nearest intersection for their workplace(s); usual grocery store; place of worship; healthcare provider; and location for leisure time besides places already listed [19, 27]. All locations were geocoded and characterized as falling within an established, emerging, or non-enclave. As a measure of enclave engagement apart from residence, we created an index representing the number of activities that took place in an enclave (whether established or emerging), with a possible range from 0 to 5; participants received a score of 5 if all five activities (employment, grocery-shopping, religious services, healthcare, leisure) took place in an enclave.

Metabolic syndrome was quantified as a score representing the number of MetS components using the harmonized definition of MetS (Table 1), with MetS indicated by the presence of at least three of the five components [28]. Our definition for elevated triglycerides included use of cholesterol-lowering medications, which can also reduce triglyceride levels; findings excluding these from our definition for elevated triglycerides were not materially different.

Sociodemographic characteristics including age, gender, marital status, length of US residence, and highest level of education were assessed at baseline. Level of acculturation was assessed at baseline and follow-up using an abridged, 11-item version of the General Ethnicity Questionnaire – American version (GEQA) [29], which assesses the respondent’s degree of engagement with American culture and activities, with higher score indicating a higher level of acculturation [30]. At baseline and follow-up, participants were also asked if they smoke now, and their average number of hours of sleep in a 24-hour period. We included other Census tract-level variables commonly used as indicators of socioeconomic disadvantage [31] as potential confounders (proportion of adults aged 25 and older with a college degree; percent of occupied housing units that were owner-occupied; percent of adults aged 18–64 years living in poverty; and median household income) using 2016–2020 data from American Community Survey 5-Year Estimates to reflect sample characteristics during the period of data collection.

### Analysis

Of the 520 participants recruited into the study, two were excluded for missing information on MetS components and two for missing covariate data, leaving a sample of 516. We used analysis-of-variance and Cochran-Mantel-Haenszel test statistics to evaluate unadjusted, bivariate associations of neighborhood type (established, emerging, and non-enclave) and categories of enclave engagement (0–1, 2, 3–5) with MetS-associated markers.

To test the hypothesis that enclave residence would be inversely associated with MetS (Hypothesis 1a), we used logistic regression analyses to model associations between neighborhood type and dichotomized MetS and MetS components variables (Table 1), using Generalized Estimating Equations (GEE) with an exchangeable correlation matrix to account for repeated measures. These models included 905 observations (516 individuals + 389 with follow-up observations) and adjusted for variables expected a priori to be associated with neighborhood type and/or CMR: age at baseline (years), gender, marital status (married or not), education level (≤ 8 years, 9–11 years, high school graduate, Bachelor’s degree or higher), length of US residence (years), acculturation (continuous GEQA score), current smoking (yes/no), hours of sleep (0–6, > 6-<8, 8+), and census tract variables mentioned above. Time (baseline or follow-up) was included as a covariate to examine change between visits. Acculturation, smoking, and sleep were included as time-dependent variables. In sensitivity analyses, to address the possibility that over-adjustment might attenuate associations for enclave residence, we excluded length of residence, acculturation, and enclave engagement as covariates. As results were not meaningfully different, estimates based on the fully adjusted model are presented.

To test the hypothesis that enclave residence would be associated with a lesser shift to MetS over time (Hypothesis 1b), we included a time × neighborhood interaction to evaluate differences by residential neighborhood. We also ran models in each of the three neighborhood types separately.

To test whether enclave engagement was associated with MetS (Hypothesis 2), models included both residential neighborhood type and time-dependent enclave engagement index to examine their independent associations with cardiometabolic outcome variables. In an additional analysis, we cross-classified participants based on both residential neighborhood type and enclave engagement index.

All analyses were conducted using SAS (version 9.4, 2013, SAS Institute, Inc., Cary, NC).

## Results

Overall, our study sample had a mean age of 52.7 years and was disproportionately female (65.7%) and married (84.7%) (Table 2). Almost half (46.3%) of individuals had eight or fewer years of education, while 5.2% had completed at least a Bachelor’s degree. Mean length of US residence was 17.6 years and was significantly higher among non-enclave residents (19.2 years) than emerging (16.3 years, *p* = 0.0041) or established (16.7 years, *p* = 0.022) enclave residents. Similarly, mean GEQA score was significantly higher among non-enclave residents (2.60) than emerging (2.42, *p* = 0.022) or established (2.43, *p* = 0.044) enclave residents. Non-enclave residents were also more likely to engage in fewer than two activities in enclaves (50.2%) than either emerging (35.7%) or established (19.8%) enclave residents (*p* < 0.0001).

With respect to MetS, 18.9% had none of the components at baseline, while almost a third (31.8%) had at least three. Elevated waist circumference was the most prevalent MetS component at baseline (58.1%), followed by elevated BP (45.5%), reduced HDL-C (32.0%), elevated triglycerides (28.7%), and elevated fasting glucose (20.0%). There were no significant differences in prevalence of MetS or its components across the three neighborhood types at baseline.

### Hypothesis 1a. Enclave Residence and Odds of MetS

In adjusted logistic regression models, established enclave residents had less than half the odds of elevated glucose relative to non-enclave residents (OR 0.45, 95% CI 0.26–0.78, *p* = 0.0047) and marginally significantly higher odds of elevated BP (OR 1.50, 95% CI 0.99–2.27, *p* = 0.056) (Table 3). Emerging enclave residents had marginally significantly lower odds of reduced HDL-C (OR 0.70, 95% CI 0.48–1.02, *p* = 0.063) and elevated BP (OR 0.69, 95% CI 0.48–1.01, *p* = 0.057) relative to non-enclave residents. No other noteworthy patterns emerged between enclave type and MetS or its components.

### Hypothesis 1b. Enclave Residence and Change in Odds of MetS

In analyses examining change over an average of 2.6 years of follow-up (Table 4), the occurrence of MetS increased in the overall sample (follow-up vs. baseline OR 1.42, 95% CI 1.04–1.95, *p* = 0.028). Increased occurrences of elevated waist circumference (follow-up vs. baseline OR 1.76, 95% CI 1.29–2.40, *p* = 0.0004) and elevated triglycerides (follow-up vs. baseline OR 1.53, 95% CI 1.11–2.10, p-0.0091) appeared to drive much of the change in MetS. The increase in occurrence of MetS was significant only for non-enclave residents (follow-up vs. baseline OR 1.69, 95% CI 1.06–2.70, *p* = 0.029) in stratified analyses, but the interaction p-value was not significant (*p* = 0.65), nor were there statistically significant differences in change in occurrence of MetS components across enclave strata (interaction p-values ranging 0.1 to 0.98).

Nevertheless, the increase in occurrence of some MetS components was notably worse in some strata. For example, residents of non-enclaves showed a greater increase in high triglycerides from baseline to follow-up (OR 2.16, 95% CI 1.32–3.55, *p* = 0.0023), and residents of established enclaves had a greater increase in high BP from baseline to follow-up (OR 2.09, 95% CI 1.05–4.17, *p* = 0.037). On the other hand, residents of emerging enclaves experienced less of an increase in elevated waist circumference over time (follow-up vs. baseline OR 1.32, 95% CI 0.75–2.31, p-0.34) than residents of either established (follow-up vs. baseline OR 2.21, 95% CI 1.11–4.41, p-0.025) or non-enclaves (follow-up vs. baseline OR 1.93, 95% CI 1.20–3.11, p-0.0068).

### Hypothesis 2. Enclave Engagement and Odds of MetS

With respect to level of engagement, participants with medium enclave engagement (2 activities in enclaves) had nearly 50% higher odds of high waist circumference relative to those with < 2 activities (OR 1.44, 95% CI 1.04–2.02, *p* = 0.031). However, overall, we saw no consistent patterns of associations for enclave engagement with MetS or MetS components (see Supplementary Table). In analyses based on joint categorization of enclave residence and engagement, participants living in either emerging or established enclaves who also engaged in at least three of the five reported activities in an enclave had the lowest odds of MetS, but this was only marginally statistically significant (OR 0.63, 95% CI 0.39–1.02, *p* = 0.06).

## Discussion

Overall, we found no clear or consistent associations of enclave residence with MetS or with change in the occurrence of MetS. Over the follow-up, the occurrence of overall MetS and of two components – high waist circumference and high triglycerides – increased significantly in the overall sample, and in stratified analyses the increase in MetS was significant only among non-enclave residents. However, interaction p-values indicated no significant differences across neighborhood types in change in MetS or any of its components. We also did not see a clear association of enclave engagement with MetS; enclave residents who engaged more frequently in activities in enclaves had 37% lower odds of MetS than non-residents who seldom visited enclaves, but this was only marginally statistically significant.

Prior research has reported mixed results when examining enclaves (or a proxy such as ethnic density or segregation) and CMR in Asian immigrants. Of eight studies that examined this question, seven examined BMI. Of these, four observed a protective effect [11, 15–17], including the only two studies conducted among Chinese samples, although in one of these, the inverse association was null after adjusting for individual and area-level covariates [11]. The only longitudinal study [12] found no association with BMI. Of the two studies that examined outcomes other than BMI, Mobley et al. [14] found a protective effect between ethnic segregation and cardiovascular risk score. In contrast, Lim et al. found no associations for residence in one of five Asian enclaves in New York City with either diabetes or hypertension, both self-reported [13]. Taken together, prior studies are divided between those showing either a protective effect or no association. However, drawing conclusions across the studies is difficult: they included different and potentially heterogeneous Asian immigrant populations; geographic scales varied from census tracts to ZIP code areas to counties or other administrative boundaries; most used self-reported outcome measures [11–13, 17, 18]; only two examined outcome measures besides BMI [13, 14]; and only one was longitudinal [12].

## New Contributions To the Literature

Our findings add to the growing number of studies that suggest ethnic enclaves are not ‘monolithically beneficial’ [7, 20, 32]. Rather, there is nuance based on the form of the enclave. For example, in the current study, results suggested that residents of established enclaves experienced more occurrences of elevated blood pressure compared to other neighborhood types. This finding may be due to higher stressors in established immigrant enclaves; for example, established enclaves may be disconnected from wider social and economic opportunities [33, 34] and sources not only of economic opportunity but also of economic exploitation [35]. Benefits of living in an enclave may also be greater for recent immigrants than for established immigrants [36].

Ours is the first study to our knowledge to investigate enclave engagement in relation to CMR in an immigrant population. While we saw no consistent pattern of associations, studies on activity spaces [19, 27] suggest that more time spent in socio-economically disadvantaged communities, whether one’s neighborhood of residence or not, are associated with higher obesity risk and poorer self-reported health. The five activities on which our measure of enclave engagement was based may not fully reflect participants’ overall engagement with an enclave. Further research might qualitatively explore immigrants’ interactions with people and spaces in enclave neighborhoods towards the development of more refined measures. Previous studies also suggest that engagement in enclaves might be associated with other health benefits – namely, mental and psychosocial wellbeing [37–39].

A limitation of our study is its sample size, which limited power to detect associations especially when stratified by neighborhood type. The relatively short follow-up (2.6 years) is another potential limitation, although it was sufficient to capture significant increases in MetS and some components in this sample; longer follow-up will provide a clearer picture of how health trajectories might differ across neighborhood types. Also worth noting is that this study was initiated just before the pandemic, and follow-up occurred after lockdown measures were relaxed. Thus, effects of enclave residence over time cannot be separated from effects of the pandemic itself. Impacts of anti-Asian violence and economic losses due to reduced patronage of enclave businesses may have been more acute for residents of established enclaves, which may have contributed to significant increases in two MetS components among established enclave residents from baseline to follow-up. Finally, our findings reflect the experience of a sample of Chinese immigrants in Philadelphia and should be confirmed in other Chinese communities and immigrant populations.

Strengths of the study include its prospective design; exploration of multiple indicators of CMR; use of measured rather than self-reported outcomes; operationalization of ethnic enclaves using empirical, census-based data supplemented with local understanding of Philadelphia neighborhoods; and focus on Chinese immigrants rather than Asian immigrants more generally.

In sum, in this sample of Chinese immigrants in Philadelphia, the occurrence of overall MetS increased over follow-up, and we identified a few differences by enclave status in changes in MetS components. However, we found no clear or consistent associations of enclave residence with change in the overall occurrence of MetS. Overall, our findings also suggest the importance of distinguishing among types of enclaves to examine potential differences in health trajectories. Two areas that merit further investigation are (1) developing more comprehensive measures of enclave engagement, and (2) identifying the physical and social resources that may support health for an immigrant community’s members, to inform effective, policy-based investments in their neighborhoods.

## Supplementary Material

supp

**Supplementary Information** The online version contains supplementary material available at https://doi.org/10.1007/s10903-025-01815-6.

## Figures and Tables

**Table 1:** Cutpoints used to define high risk for metabolic syndrome components and related measures [28]

Measure	Cutpoint
Metabolic syndrome	Meeting 3 + component criteria for metabolic syndrome
Elevated waist circumference	Women ≥ 80 cm, men ≥ 85 cm^a^
Elevated triglycerides	≥ 150 mg/dL or self-reported drug treatment for high cholesterol
Reduced HDL-C	Women < 50 mg/dL, men < 40 mg/dL
Elevated blood pressure	Systolic ≥ 130 and/or diastolic ≥ 85 mm Hg or self-reported antihypertensive drug treatment
Elevated fasting glucose	≥ 100 mg/dL or self-reported doctor-diagnosed elevated glucose

a Cutpoints suggested for Chinese population from Working Group on Obesity in China [40], cited in [28]

**Table 2:** Descriptive characteristics of study sample at baseline

	Overall (n = 516)	Non-Enclave (n = 219)	Emerging (n = 171)	Established (n = 126)	*p*-value^a^
Mean (SD) age (years)	52.7 (7.7)	52.8 (8.0)	53.1 (6.8)	51.9 (8.2)	0.43
Male (%)	34.3	38.4	33.3	28.6	0.17
Married (%)	84.7	85.4	86.6	81.0	0.39
Education Level (%)					0.17
≤8 years	46.3	44.3	44.4	52.4	
9–11 years or vocational/technical school	17.4	16.9	21.6	12.7	
High school completion, some college, or Associates Degree	31.0	32.0	31.6	28.6	
Bachelor’s or graduate degree	5.2	6.9	2.3	6.4	
Mean (SD) General Ethnicity Questionnaire score	2.50 (0.75)	2.60 (0.75)	2.42 (0.73)	2.43 (0.76)	**0.035**
Mean (SD) length of US residence (years)	17.6 (9.8)	19.2 (10.3)	16.3 (8.9)	16.7 (10.0)	**0.0075**
Enclave engagement index (%)					**< 0.0001**
0–1	38.0	50.2	35.7	19.8	
2	34.3	29.2	40.9	34.1	
3–5	27.7	20.6	23.4	46.0	
Current Smoker (%)	7.0	8.7	8.2	2.4	0.066
Hours of sleep (%)					0.46
0–6	24.2	22.4	24.0	27.8	
>6- <8	34.5	32.0	37.4	34.9	
≥8	41.3	45.7	38.6	37.3	
Elevated waist circumference	58.1	58.0	59.7	56.4	0.85
Elevated triglycerides	28.7	28.8	31.0	25.4	0.57
Reduced HDL-C	32.0	34.7	30.4	29.4	0.51
Elevated blood pressure	45.5	47.5	42.1	46.8	0.54
Elevated fasting glucose	20.0	22.4	21.1	14.3	0.18
Number of metabolic syndrome components (%)					0.57
0	18.9	18.3	19.3	19.1	
1	24.8	24.2	24.6	26.2	
2	24.6	23.3	24.0	27.8	
3	18.8	17.8	19.9	19.1	
4	11.1	15.1	9.4	6.4	
5	1.9	1.4	2.9	1.6	
Metabolic syndrome (≥ 3 components) (%)	31.8	34.3	32.2	27.0	0.38

a P-value for difference in distribution of across neighborhoods using Cochran-Mantel-Haenszel chi-square test or analysis-of-variance for continuous variables

**Table 3:** Adjusted^a^ odds ratios and 95% confidence intervals for associations of residential neighborhood type with metabolic syndrome and its components (*n* = 905 observations from 516 participants). Boldface indicates statistically significant odds ratios

	Non-enclave (reference)	Emerging enclave	Established enclave
Elevated waist circumference	1.00	0.91 (0.62–1.32)	0.95 (0.63–1.43)
p-value		0.60	0.80
Elevated triglycerides	1.00	1.04 (0.70–1.53)	0.89 (0.58–1.37)
p-value		0.86	0.60
Reduced HDL-C	1.00	0.70 (0.48–1.02)	1.10 (0.72–1.67)
p-value		0.063	0.66
Elevated blood pressure	1.00	0.69 (0.48–1.01)	1.50 (0.99–2.27)
p-value		0.057	0.056
Elevated fasting glucose	1.00	1.33 (0.85–2.08)	**0.45** **(0.26–0.78)**
p-value		0.22	0.0047
Metabolic syndrome	1.00	0.80 (0.55–1.18)	0.89 (0.58–1.35)
p-value		0.27	0.58

a Models included residential neighborhood type (non-enclave, emerging enclave, established enclave), enclave engagement (0–1, 2, or 3–5 activities conducted in enclave), time (baseline or follow-up), age at baseline (years), gender, marital status (married or not), education level (≤ 8 years, 9–11 years, high school graduate, Bachelors degree or higher), length of residence in the US (years), acculturation level (continuous GEQA score), current smoking (yes or no), hours of sleep (0–6, > 6-<8, 8+), census tract variables for college degree, median household income, poverty, homes owner-occupied. Models included baseline and follow-up timepoints

**Table 4:** Adjusted^a^ odds ratios and 95% confidence intervals for change in metabolic syndrome and its components comparing follow-up vs. baseline (reference), for overall pooled sample and stratified by residential neighborhood type (*n* = 905 observations from 516 participants). Boldface indicates statistically significant odds ratios

	All	Stratified		
		Non-Enclave	Emerging	Established
Elevated waist circumference	**1.76 (1.29–2.40)**	**1.93 (1.20–3.11)**	1.32 (0.75–2.31)	**2.21 (1.11–4.41)**
p-value	**0.0004**	**0.0068**	0.34	**0.025**
Interaction p-value			0.28	
Elevated triglycerides	**1.53 (1.11–2.10)**	**2.16 (1.32–3.55)**	1.16 (0.64–2.11)	1.30 (0.65–2.63)
p-value	**0.0091**	**0.0023**	0.63	0.46
Interaction p-value			0.18	
Reduced HDL-C	1.05 (0.77–1.44)	1.09 (0.68–1.73)	1.08 (0.59–1.97)	0.95 (0.46–1.96)
p-value	0.75	0.73	0.79	0.89
Interaction p-value			0.98	
Elevated blood pressure	1.23 (0.91–1.68)	1.26 (0.80–1.98)	0.90 (0.49–1.64)	**2.09 (1.05–4.17)**
p-value	0.18	0.33	0.72	**0.037**
Interaction p-value			0.096	
Elevated fasting glucose	1.25 (0.86–1.80)	1.22 (0.71–2.10)	1.44 (0.75–2.80)	1.13 (0.43–2.98)
p-value	0.24	0.48	0.28	0.80
Interaction p-value			0.29	
Metabolic syndrome	**1.42 (1.04–1.95)**	**1.69 (1.06–2.70)**	1.31 (0.71–2.40)	1.34 (0.66–2.74)
p-value	**0.028**	**0.029**	0.39	0.42
Interaction p-value			0.65	

a Models included enclave engagement (0–1, 2, or 3–5 activities conducted in enclave), time (baseline or follow-up), age at baseline (years), gender, marital status (married or not), education level (≤ 8 years, 9–11 years, high school graduate, Bachelors degree or higher), length of residence in the US (years), acculturation level (continuous GEQA score), current smoking (yes or no), hours of sleep (0–6, > 6-<8, 8+), census tract variables college degree, median household income, poverty. and homes owner-occupied. Model for overall sample also included residential neighborhood type (non-enclave, emerging enclave, established enclave)
