# Supplementary material for: Ethnic Enclaves and Metabolic Syndrome in Chinese Immigrants in Philadelphia
Source: J Immigr Minor Health. 2025 Nov 20;28(3):627–36. doi: 10.1007/s10903-025-01815-6 (PMC13215018; doi:10.1007/s10903-025-01815-6)
Supplement: Supplementary file 1 — Supplementary Material 1 [file 10903_2025_1815_MOESM1_ESM.docx]

Supplemental table. Adjusted^a^ odds ratios and 95% confidence intervals for associations of enclave engagement and variable combining enclave residence and engagement with change in metabolic syndrome and its components (n=905 observations from 516 participants). Boldface indicates statistically significant odds ratios.

|  | Number of activities in enclave (relative to 0-1 activities) | | Combined enclave residence and number of activities in enclave (relative to non-enclave residents with 0-2 enclave activities) | |
| --- | --- | --- | --- | --- |
|  | 2 | 3-5 | Non-enclave residents with 3-5 enclave activities OR enclave residents with 0-2 enclave activities | Enclave residents with 3-5 enclave activities |
| Elevated waist circumference | **1.44 (1.03-2.02)** | 1.21 (0.81-1.80) | 1.04 (0.76-1.44) | 0.92 (0.59-1.43) |
| p-value | **0.03** | 0.35 | 0.79 | 0.71 |
| Elevated triglycerides | 1.26 (0.89-1.76) | 1.02 (0.67-1.55) | 1.06 (0.77-1.47) | 0.84 (0.52-1.35) |
| p-value | 0.19 | 0.93 | 0.72 | 0.47 |
| Reduced HDL-C | 1.18 (0.85-1.64) | 0.76 (0.50-1.15) | 0.91 (0.66-1.25) | **0.58 (0.36-0.94)** |
| p-value | 0.32 | 0.19 | 0.55 | **0.027** |
| Elevated blood pressure | 0.89 (0.64-1.24) | 0.83 (0.55-1.24) | 1.01 (0.74-1.39) | 0.87 (0.56-1.36) |
| p-value | 0.50 | 0.36 | 0.93 | 0.53 |
| Elevated fasting glucose | 1.19 (0.81-1.77) | 1.10 (0.66-1.80) | 0.92 (0.63-1.34) | 0.78 (0.45-1.35) |
| p-value | 0.38 | 0.72 | 0.66 | 0.38 |
| Metabolic syndrome | 1.15 (0.83-1.61) | 0.84 (0.55-1.29) | 0.91 (0.66-1.26) | 0.63 (0.39-1.02) |
| p-value | 0.40 | 0.43 | 0.58 | 0.060 |

^a^ Models for both number of activities in enclave and combined residence-engagement variable included time (baseline or follow-up), age at baseline (years), gender, marital status (married or not), education level (≤8 years, 9-11 years, high school graduate, Bachelors degree or higher), length of residence in the US (years), acculturation level (continuous GEQA score), current smoking (yes or no), hours of sleep (0-6, >6-<8, 8+), census tract variables for college degree, median household income, poverty, homes owner-occupied. Models for number of activities in enclave also included residential neighborhood type (non-enclave, emerging enclave, established enclave).
